# Effectiveness and safety of acupoint catgut embedding for patients with mild psoriasis and overweight: study protocol of a multicenter double-blind randomized controlled trial

**DOI:** 10.3389/fmed.2026.1796602

**Published:** 2026-03-19

**Authors:** Xuanchun Zhang, Di Long, Junchen Chen, Ziwan Lei, Jing Hu, Yawen Chen, Biying Wang, Yehong Kuang, Haizhen Wang

**Affiliations:** 1 Department of Dermatology, The Second Affiliated Hospital of Hunan University of Chinese Medicine, Changsha, China; 2 Department of Dermatology, Xiangya Hospital, Central South University, Changsha, China

**Keywords:** acupoint catgut embedding, comorbidity, obesity, overweight, perineal trauma, psoriasis

## Abstract

**Background:**

Psoriasis is a systemic inflammatory disease frequently comorbid with obesity, as both conditions share common pathogenic pathways. Acupoint catgut embedding (ACE), a sustained-release form of acupuncture, has demonstrated potential in modulating both immune and metabolic responses. This trial aims to evaluate the efficacy, safety, and underlying mechanisms of ACE in patients with mild psoriasis and concurrent overweight/obesity.

**Methods:**

This is a multicenter, randomized, double-blind, sham-controlled trial. Participants aged ≥ 18 years with mild plaque psoriasis (PASI< 3, BSA < 3%) and overweight (BMI ≥ 24 kg/m^2^) will be randomized (1:1) to receive either verum ACE at 14 specific acupoints or a sham procedure. Interventions will be administered every 2 weeks for 8 weeks. The primary endpoints are the changes in PASI score and BMI from baseline to week 8. Secondary outcomes include the Dermatology Life Quality Index (DLQI), waist circumference (WC), lipid profiles, and systemic inflammatory markers. The safety of the procedure will be monitored through the recording of adverse events and conducting laboratory tests. Participants will be followed through week 32 to evaluate long-term therapeutic durability.

**Discussion:**

This trial will provide high-quality evidence regarding the “dual-action” potential of ACE in simultaneously improving psoriatic lesions and metabolic dysfunction, establishing its role as an integrative therapy for this specific patient phenotype.

**Clinical trial registration:**

https://itmctr.ccebtcm.org.cn/, identifier ITMCTR2025002460.

## 1 Introduction

Psoriasis is a chronic, immune-mediated inflammatory skin disorder driven by a complex interplay of genetic and environmental factors. Beyond its cutaneous manifestations, psoriasis is increasingly recognized as a systemic disease, frequently co-occurring with metabolic syndrome (MetS) in 20 to 50% of patients (1, 2). Key components of MetS—including obesity, dyslipidemia, and impaired glucose metabolism—are not merely comorbidities but are fundamentally linked to the disease’s severity (3–5). Evidence shows that the Psoriasis Area, Severity Index (PASI) scores positively correlate with body weight, cholesterol levels, and glycemic markers (6). Crucially, these metabolic abnormalities often persist after skin clearance, acting as long-term prognostic markers that can trigger future relapses (7, 8).

Central to this systemic interplay is obesity, which serves as a potent aggravating factor for psoriatic inflammation. Adipose tissue in obese individuals acts as an active endocrine organ, secreting pro-inflammatory cytokines such as leptin, IL-6, and TNF-*α*, while directly fuel the Th17/IL-17/IL-23 axis—the primary driver of psoriatic lesions (9–13). This creates a self-perpetuating “psoriatic-metabolic” inflammatory loop. While clinical evidence underscores that weight loss can significantly improve psoriasis outcomes (14, 15), standard systemic treatments often fail to address this metabolic driver. For instance, while biologics offer rapid skin clearance, their impact on metabolic parameters like BMI and insulin resistance remains inconsistent and lacks confirmation from large-scale prospective trials (16).

To bridge this therapeutic gap, Acupoint Catgut Embedding (ACE), emerges as a promising integrative strategy. As a sustained-release form of acupuncture, ACE involves embedding absorbable sutures into specific acupoints to provide continuous stimulation (17–22). By embedding absorbable catgut sutures at specific acupoints, ACE provides continuous and sustained stimulation to elicit therapeutic responses, thereby achieving long-lasting effects (23). Years of research have confirmed its efficacy and safety. In particular, the application of ACE for the treatment of obesity is supported by extensive clinical observations and mechanistic studies (24). Li et al. found that ACE can improve body composition, including reductions in body mass index (BMI) and waist-to-hip ratio (WHR) through appetite regulation, while also ameliorating metabolic parameters such as blood lipid and glucose levels (25–28). More importantly, its mechanism closely with the needs of psoriatic patients: ACE modulates chronic low-grade inflammation by downregulating IL-6, TNF-*α*, and MCP-1, while simultaneously ameliorating leptin resistance and enhancing insulin sensitivity (29, 30).

Despite its therapeutic potential, the clinical adoption of ACE for psoriasis remains hindered by a lack of high-quality evidence. Current research is predominantly limited to small-scale case series or trials with methodological inconsistencies. There is an urgent need for robust, multicenter randomized controlled trials (RCTs) to definitively establish the efficacy and safety of ACE in this specific patient population and to elucidate its underlying biological mechanisms.

This multicenter, double-blind, randomized controlled trial aims to evaluate the efficacy of ACE compared to a sham procedure in patients with mild psoriasis and overweight/obesity. We hypothesize that ACE will demonstrate superior efficacy in reducing both psoriasis severity and metabolic dysfunction, providing a novel integrative strategy for this specific patient phenotype.

## 2 Methods and design

### 2.1 Study setting and recruitment

**Figure 1:**
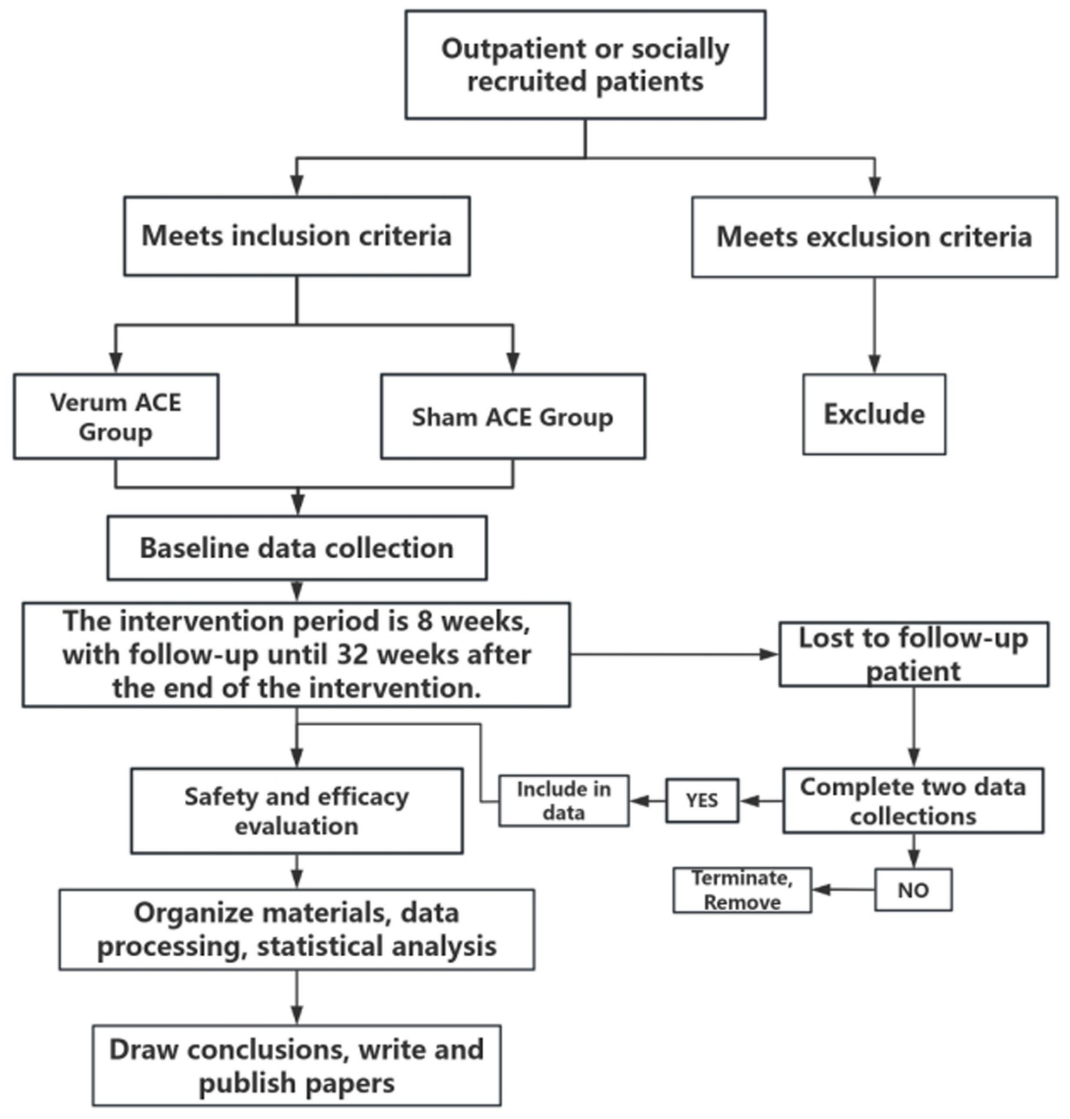
Flow diagram of the study design.

This multicenter double-blind randomized controlled trial employs a two-arm, 1:1 allocation design. Conducted from January 2026 to December 2027, the study will recruit participants primarily from the dermatology outpatient clinics of two tertiary hospitals—the Second Affiliated Hospital of Hunan University of Chinese Medicine and Xiangya Hospital, Central South University, both are tertiary hospitals, supplemented by community-based recruitment (like publishing recruitment advertisements through online platforms). The trial procedure is summarized in Figure 1. The study protocol was developed in accordance with the Interventional Trials (SPIRIT) guidelines (31) and STRICTA guidelines (32).

### 2.2 Randomization and blinding

Participants will be randomly assigned to either the verum ACE group or the sham ACE group in a 1:1. A computer-based central randomization system, managed by an independent statistician not involved in patient recruitment or intervention, will be used to generate the allocation sequence (33).

Participants, data collectors, data analysts, and recruiting physicians will be blinded to group allocation. To ensure blinding, the recruitment physician will be responsible for screening potential participants, obtaining informed consent, and completing the randomization procedure, but will have no further involvement in the trial. Following randomization, operators will use delivery devices pre-loaded according to the allocation codes, such that the presence or absence of the absorbable suture remains concealed. Except for the actual embedding of the catgut, all procedural steps—acupoint localization, skin disinfection, and needling technique—will be strictly standardized across both groups to minimize performance bias (34–37). Data collectors, who will assess clinical outcomes, and the statistician performing the final analysis will be also blinded to group allocation. This multi-tiered blinding strategy ensure that only the third-party researcher preparing the kits is aware of the allocation sequence. At trial completion, a blinding assessment will be conducted among participants and practitioners, and the accuracy of their group guesses will be tested against the 50% chance level using a chi-square test. This rigorous design ensures that treating physicians, data collectors, statisticians, operators, and patients remain blinded throughout the study. The trial is registered with the International Traditional Medicine Clinical Trial Registration Platform (Registration No. ITMCTR2025002460).

### 2.3 Rationale for the sham control

A sham procedure was selected as the control for the following reasons:

(1) *Methodological rigor*: As a double-blind, randomized controlled trial, a sham procedure is essential. It matches the verum ACE in all visible and sensory aspects, ensuring effective participant blinding and controlling for non-specific effects (e.g., placebo, natural disease variation). This allows for an objective assessment of the specific therapeutic efficacy attributable to the catgut embedding.
(2) *Establishing clinical superiority*: Current standard treatments for mild psoriasis have limitations, including side effects and lack of efficacy for obesity. This trial aims to prove that ACE offers a specific therapeutic benefit, which necessitates comparison against a non-active intervention is necessary to establish its distinct clinical value and therapeutic advantage.

### 2.4 Research objectives

- *Primary objective*: to evaluate the efficacy of ACE therapy as an adjunctive treatment for improving the PASI score and reducing BMI after 8 weeks in patients with mild psoriasis and overweight.
- *Secondary objectives*: (1) to assess the changes in secondary efficacy indicators of psoriasis, BMI, waist circumference (WC), WHR and metabolic-related indicators, etc. measures from baseline to week 8. (2) to document the incidence of adverse events (AEs) throughout the study period. (3) to determine the psoriasis recurrence rate during the follow-up period.

### 2.5 Inclusion criteria

(1) Aged 18 years or older, male or female.
(2) Clinical diagnosis of plaque psoriasis with blood stasis syndrome confirmed by traditional Chinese medicine criteria.
(3) Mild psoriasis, defined as PASI score < 3 or BSA < 3%.
(4) Overweight, defined as BMI ≥ 24 kg/m^2^.
(5) Ability to comprehend the study objectives, procedures, potential benefits, and risks.
(6) Willingness to complete all scheduled visits and provide truthful feedback.
(7) Provision of voluntarily signed informed consent.

### 2.6 Exclusion criteria

(1) Recent psoriasis treatments, including systemic therapy (e.g., cyclosporine, methotrexate), phototherapy, traditional Chinese medicine, or small-molecule targeted agents within 1 month or biologic agents within 3 months prior to enrollment.
(2) Presence of clinically significant comorbidities, such as coagulation disorders, infectious diseases, psychiatric conditions, autoimmune diseases, malignancies, or major organ dysfunction.
(3) Significant weight fluctuation or notable body shape alteration within the past 3 months.
(4) Active weight-loss interventions (e.g., dietary control, supplements, regular exercise) accompanied by weight reduction within the past 3 months.
(5) History of keloid formation or hypertrophic scarring.
(6) Participation in any other clinical trial within the past 6 months.
(7) Pregnancy, lactation, or planned pregnancy during the study period.
(8) Patients with needle phobia.
(9) Inability or unwillingness to comply with the intervention, attend scheduled follow-ups, or complete required study procedures.
(10) Any other condition considered by the investigators to potentially affect the validity of the study or the safety of the participant.

### 2.7 Termination criteria

The entire study will be terminated if:

(1) Serious safety concerns are identified that preclude further participation.
(2) The intervention demonstrates negligible efficacy or lacks clinical value.
(3) Major protocol deviations occur that compromise data validity.
(4) The sponsor terminates the study for administrative or financial reasons.
(5) Regulatory authorities mandate study discontinuation.

### 2.8 Withdrawal criteria

Participation will be discontinued if any of the following criteria are met:

(1) Failure to attend scheduled outpatient follow-ups on time.
(2) Occurrence of serious adverse reactions.
(3) Changes in the participant’s medical condition that render continued participation inappropriate.
(4) The participant or their legal guardian requests to stop.

#### 2.8.1 Data handling for terminated cases

For participants who are withdrawn, all collected data will be retained in the case report forms. The last available observation will be used as the final outcome for both efficacy and safety analyses.

### 2.9 Diagnostic criteria

The diagnosis of plaque psoriasis is prepared with reference to the “Chinese Psoriasis Diagnosis and Treatment Guidelines (2023 Edition)” (38). Diagnosis is based on characteristic features including well-demarcated, dark-red plaques with silvery-white scaling, classic signs (e.g., candle-grease sign, Auspitz’s sign), and a chronic, relapsing course.

### 2.10 Diagnostic criteria for blood stasis pattern in psoriasis

According to the “2017 Consensus on the Treatment of Psoriasis with Traditional Chinese Medicineissued” by the Chinese Society of Dermatology (39), the diagnosis of Blood Stasis Syndrome can be established when two primary symptoms (chronic, recurrent lesions with dark-red or violaceous coloration) and at least two secondary symptoms (dark-colored menstrual blood or clots in female or experience localized pain) are present, supported by tongue and pulse signs (purple or dark tongue body with ecchymosis or petechiae).

### 2.11 Sample size calculations

Based on our pilot data, the verum ACE achieved an improvement rate of 60%. Since no sham-controlled acupuncture trials for psoriasis are available, we derived the sham group response rate from the placebo effect observed in psoriasis pharmacological trials, which was approximately 25% as reported in the literature (40). Sample size estimation was performed using the standard formula for comparing two independent proportions (two-sided test, *α* = 0.05, power = 80%):


$$n1=n2=\frac{{\left(Z_{1-\alpha /2}+Z_{1-\beta }\right)}^{2}\times [p_{1}(1-p_{1})+p_{2}(1-p_{2})]}{{\left(p_{1}-p_{2}\right)}^{2}}$$

Where p_1_ = 0.60, p_2_ = 0.25, Z_1 − α/2_ = 1.96, and Z_1 − β_ = 0.84. The calculated sample size was 28 participants per group. After adjusting for a 20% dropout rate, the final sample size was determined to be 35 participants per group (total 70 participants).

### 2.12 Intervention

All participants will receive standardized baseline care. For skin lesions and xerosis, urea vitamin E cream (Fuyuan Pharmaceutical Co., Ltd., National Medicine Permit No. H34022160) will be applied as a moisturizer. Additionally, all patients will undergo health education regarding lifestyle and dietary modifications, along with training in daily skin care. To enhance adherence and objectively monitor compliance, researchers will add all participants to a WeChat health management group.

Participants will be instructed to photograph their daily meals; the images will be analyzed via a dedicated mobile application to estimate caloric intake. Daily step counts will be automatically recorded and tracked through the WeChat sports mini-program.

Throughout the trial, participants may continue their usual medications for comorbidities (e.g., diabetes, hyperlipidemia). We will require them to maintain the same types and doses of their underlying disease medications during the trial, with any changes in medication recorded at each visit. However, the use of any treatments specifically targeting psoriasis is prohibited.

(1) Experimental group:

In addition to conventional care, patients in the verum group will receive true catgut embedding acupuncture (needle insertion followed by thread embedding), at the following points: CV12 (Zhongwan), CV4 (Guanyuan), SP15 (Daheng), ST25 (Tianshu), ST36 (Zusanli), BL20 (Pishu), BL18 (Ganshu), and SP10 (Xuehai). All points are needled bilaterally except for CV 12 and CV 4. These acupoints were selected based on a comprehensive review of prior literature and established clinical expertise. This combination is designed to simultaneously address psoriatic inflammation and underlying metabolic dysfunction. Previous studies suggest that points such as ST25 (Tianshu), SP15 (Daheng), CV12 (Zhongwan), ST36 (Zusanli), and BL20 (Pishu) can help improve central obesity and gastrointestinal function (41, 42), while SP10 (Xuehai) is traditionally used to activate blood circulation and resolve stasis, and BL18 (Ganshu) is employed to promote blood circulation and regulate qi.

**Figure 2:**
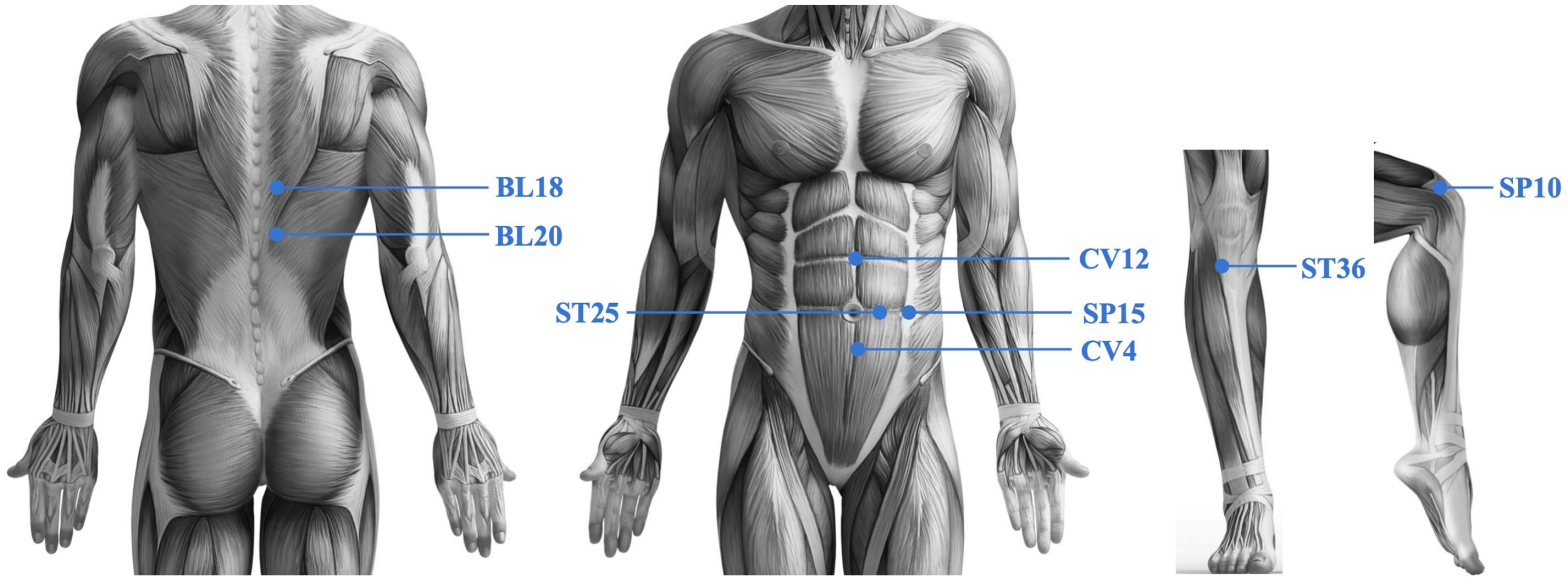
Acupoint locations for ACE of mild psoriasis combined with overweight.

The acupoints were localized according to the World Health Organization Standard Acupuncture Point Locations. All acupuncture point locations are shown in Figure 2.

*CV12*: On the anterior midline, 4 cun superior to the umbilicus.
*CV4*: On the anterior midline, 3 cun inferior to the umbilicus.
*SP15*: 4 cun lateral to the umbilicus, level with the umbilicus.
*ST25*: 2 cun lateral to the umbilicus, level with the umbilicus.
*ST36*: On the anterior aspect of the leg, 3 cun inferior to the lateral depression of the knee joint (Lateralmere, ST35) and one finger-breadth lateral from the anterior crest of the tibia.
*BL20*: On the back, at the level of the lower border of the spinous process of the 11th thoracic vertebra (T11), 1.5 cun lateral to the posterior midline.
*BL18*: On the back, at the level of the lower border of the spinous process of the 9th thoracic vertebra (T9), 1.5 cun lateral to the posterior midline.
*SP10*: On the medial aspect of the thigh, with the knee flexed, 2 cun superior to the medial superior border of the patella, on the bulge of the medial portion of the quadriceps muscle.

The “cun” is a relative unit of measurement in acupuncture, individually defined for each participant. One cun is equivalent to the width of the participant’s interphalangeal joint of the thumb.

(2) Control group:

To ensure rigorous blinding and control for the placebo effect, participants in the control group will receive a sham ACE procedure. This procedure is designed to be indistinguishable from the verum ACE in terms of visual, tactile, and procedural experience: A third-party researcher provided the acupuncturist with a pre-packed kit containing 14 disposable embedding needles corresponding to the patient’s randomization number. The acupuncturist, who remained blinded to the group assignment, performed the procedure directly using the provided kit. Although the sham procedure mimics the motions of needle insertion, thread embedding, and needle withdrawal, no absorbable suture is actually embedded.

### 2.13 The procedure of acupuncture thread embedding

To maintain allocation concealment, a third-party researcher not involved in the clinical assessment will prepare the needles according to the randomization schedule all procedures will be performed in accordance with the “Acupuncture Technical Operation Standards, Part 10: Acupoint Embedding (43).” The intervention will be conducted in a temperature-controlled room under strict aseptic conditions.

**Figure 3:**
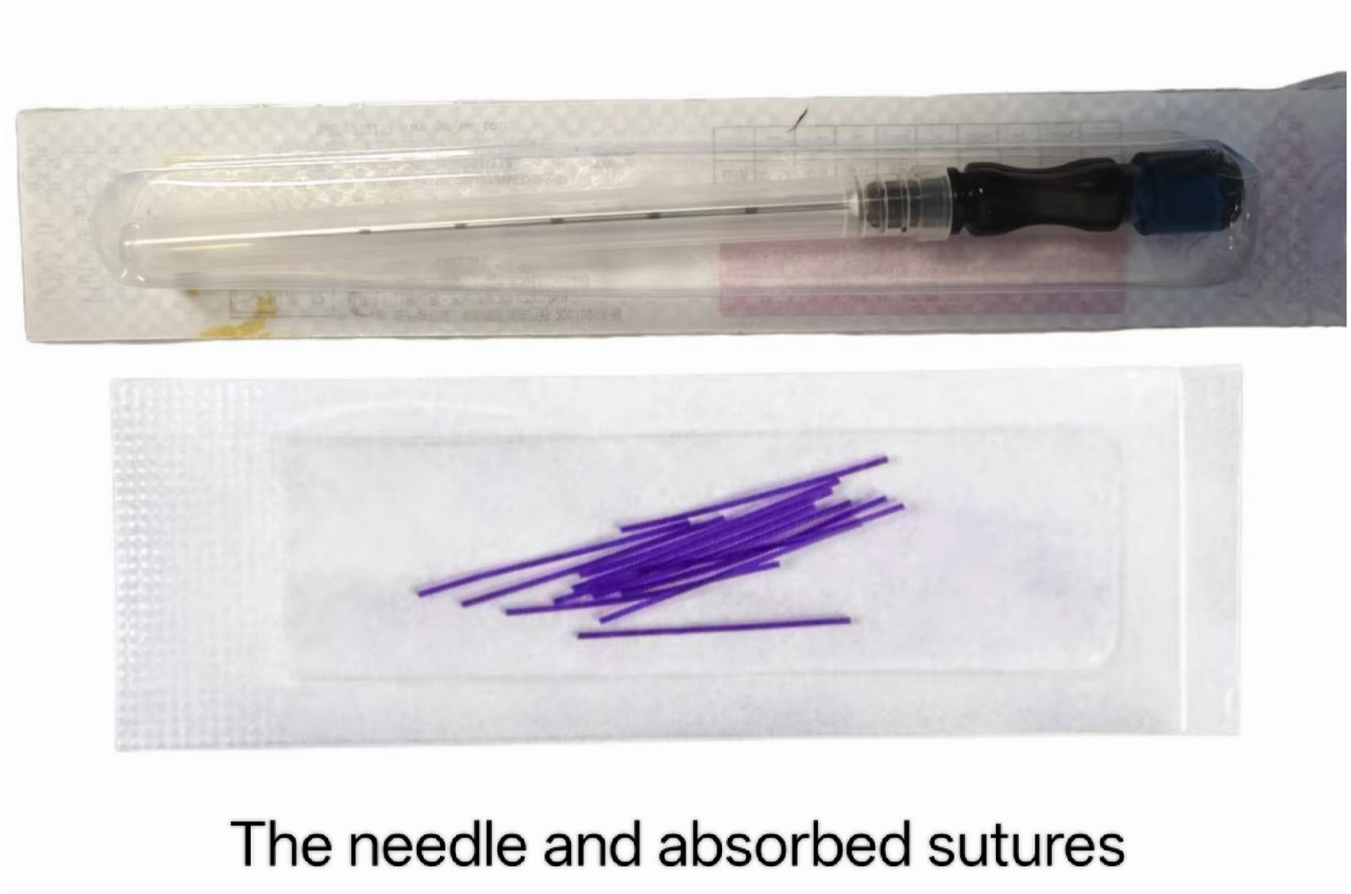
ACE needle and absorbed sutures.

Practitioners will adhere to standard surgical hand-hygiene protocols and wear sterile cap and mask. For the verum group, opaque, disposable ACE needles (0.7 × 60 mm; Su Food and Drug Administration Medical Device Manufacturing License 20010244) will be preloaded with a 2 cm segment of absorbable surgical suture (Zhejiang Food and Drug Administration Medical Device Manufacturing License 20110110). See Figure 3.

For the verum group, the needle will be inserted manually into the selected acupoints. The ST25, SP15, CV12 and ST36 will be punctured perpendicularly, with an insertion depth of approximately 25–35 mm. The BL18 and BL20 will be punctured at a 45°angle to the skin, with an insertion depth of about 15–20 mm. Once the acupoint sensation is achieved, the practitioner withdraws the needle while pushing the absorbable thread into the muscle layer using the needle core. Immediately following, needle withdrawal, the puncture sites will be promptly compressed with sterile dry cotton swabs to prevent bleeding. The placement of the thread depends on the depth of the needling, and the practitioner will not feel any resistance while inserting the absorbable thread. The treatment will be administered once every 2 weeks for a total of four sessions, lasting 8 weeks (24, 34).

For the sham group: using the same disposable needles (with the catgut suture pre-removed), the practitioner performed an identical procedure—insertion into the muscle layer followed by a simulated “pushing-withdrawing” action—ensuring the same tactile experience for participants as the verum group, with the sole difference being the absence of actual suture embedding.

A sham procedure was selected as the control for​ the following reasons: (1) to ensure rigorous trial design: as a double-blind, randomized controlled trial, a sham procedure is essential. It matches the verum ACE in every visible aspect, ensuring effective participant blinding and controlling for non-specific effects (e.g., placebo, natural disease variation). This allows for an objective evaluation of the specific therapeutic effect attributable to the catgut embedding itself. (2) to demonstrate specific efficacy beyond standard care limitations: Current standard treatments for mild psoriasis have limitations, including side effects and lack of efficacy for obesity. This trial aims to prove that ACE offers a specific therapeutic benefit, which necessitates comparison against a non-active intervention to establish its distinct clinical value.

Participation will be discontinued if any of the following criteria are met: (1) failure to attend scheduled outpatient follow-ups on time; (2) occurrence of serious adverse reactions; (3) changes in the participant’s medical condition that render continued participation inappropriate; (4) the participant or their legal guardian requests to stop.

### 2.14 Participant management and safety

Participants are encouraged to maintain open communication with the research team, and all inquiries will be addressed promptly throughout the study. To facilitate adherence to the visit schedule, transportation reimbursements will be provided for all study-related appointments. Following the intervention period, all participants will remain under routine clinical observation by the research team. Participants will be instructed to promptly report any discomfort or potential adverse reactions to the coordinator. Upon such notification, the research team will evaluate the condition and arrange appropriate post-trial care, including specialist consultations at no cost to the participant. All adverse events will be managed in accordance with standard clinical protocols, and all trial-related medical expenses will be fully covered by the study.

### 2.15 Data collection procedures

The study comprises an 8 weeks intervention period followed by a 32-week follow-up phase. During the intervention, data collection and clinical assessments will occur biweekly. Post-intervention follow-up evaluations are scheduled every four weeks through week 24, with a final assessment at week 32.

**Figure 4:**
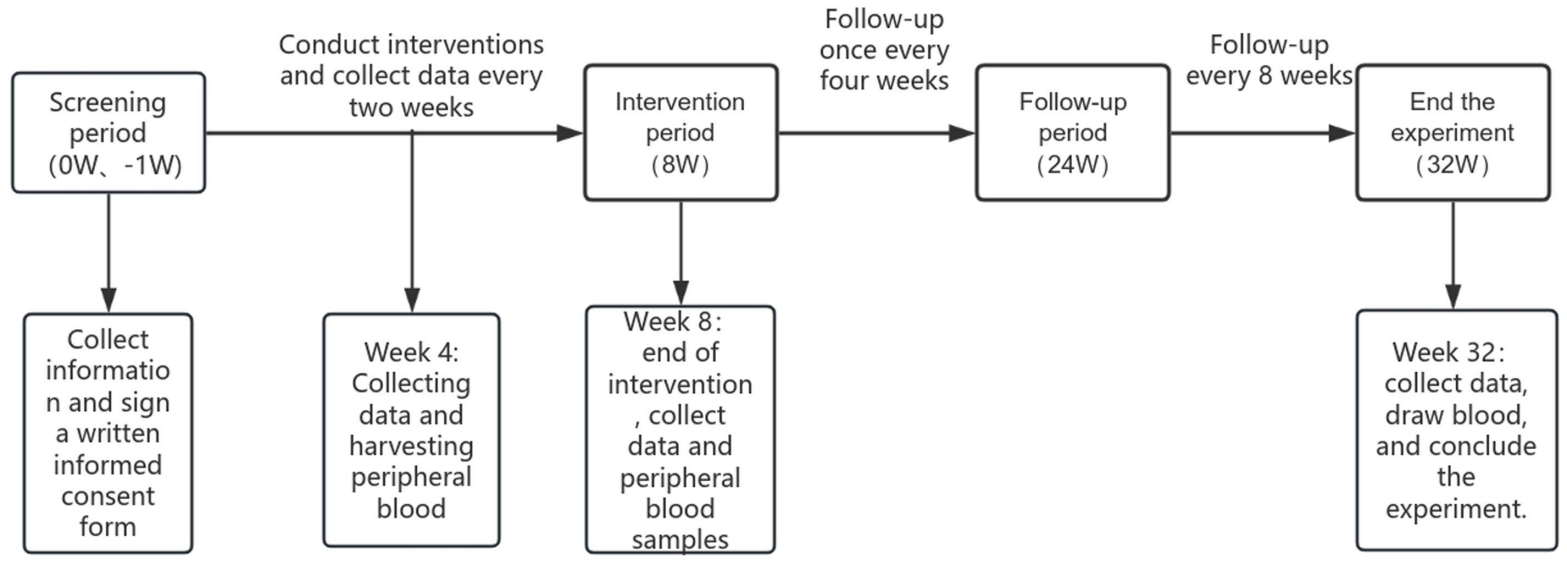
Data node collection diagram.

To explore the biological underpinnings of the therapeutic response, peripheral venous blood samples will be collected at baseline (week 0), mid-intervention (week 4), end-of-treatment (week 8), and at the final follow-up (week 32). These supplementary samples will support exploratory multi-omics analyses, including single-cell sequencing, untargeted metabolomics, and proteomics (Figure 4). A comprehensive schedule of all assessment items is provided in Table 1.

**Table 1:** The complete set of assessment items.

Project	0 W	4 W	8 W	12 W	16 W	24 W	32 W
PASI	**×**	**×**	**×**	**×**	**×**	**×**	**×**
BSA	**×**	**×**	**×**	**×**	**×**	**×**	**×**
PGA	**×**	**×**	**×**	**×**	**×**	**×**	**×**
Itch NRS score	**×**	**×**	**×**	**×**	**×**	**×**	**×**
DLQI	**×**	**×**	**×**	**×**	**×**	**×**	**×**
BMI	**×**	**×**	**×**	**×**	**×**	**×**	**×**
WC	**×**	**×**	**×**	**×**	**×**	**×**	**×**
WHR	**×**	**×**	**×**	**×**	**×**	**×**	**×**
Blood pressure	**×**	**×**	**×**	**×**	**×**	**×**	**×**
Traditional Chinese Medicine Syndrome Scoring	**×**	**×**	**×**				
ESR, CRP	**×**	**×**	**×**				**×**
Lipid profile	**×**	**×**	**×**				**×**
Blood glucose	**×**	**×**	**×**				**×**
Blood uric acid	**×**	**×**	**×**				**×**
Peripheral blood sample	**×**	**×**	**×**				**×**

If female, additional baseline blood HCG tests are required.

We will assess the safety of acupoint embedding therapy through the collection of biological sample information, including but not limited to general data, blood pressure, heart rate, and blood sample collection (Table 2).

**Table 2:** Safety indicators.

Project	0 W	4 W	8 W	12 W	16 W	24 W	32 W
Basic information	**×**						
Medical information	**×**						
Complete blood count	**×**		**×**				
Liver function, kidney function	**×**		**×**				
Electrocardiogram (ECG)	**×**		**×**				
AEs		**×**	**×**	**×**	**×**	**×**	**×**

### 2.16 Participant timeline

**Figure 5:**
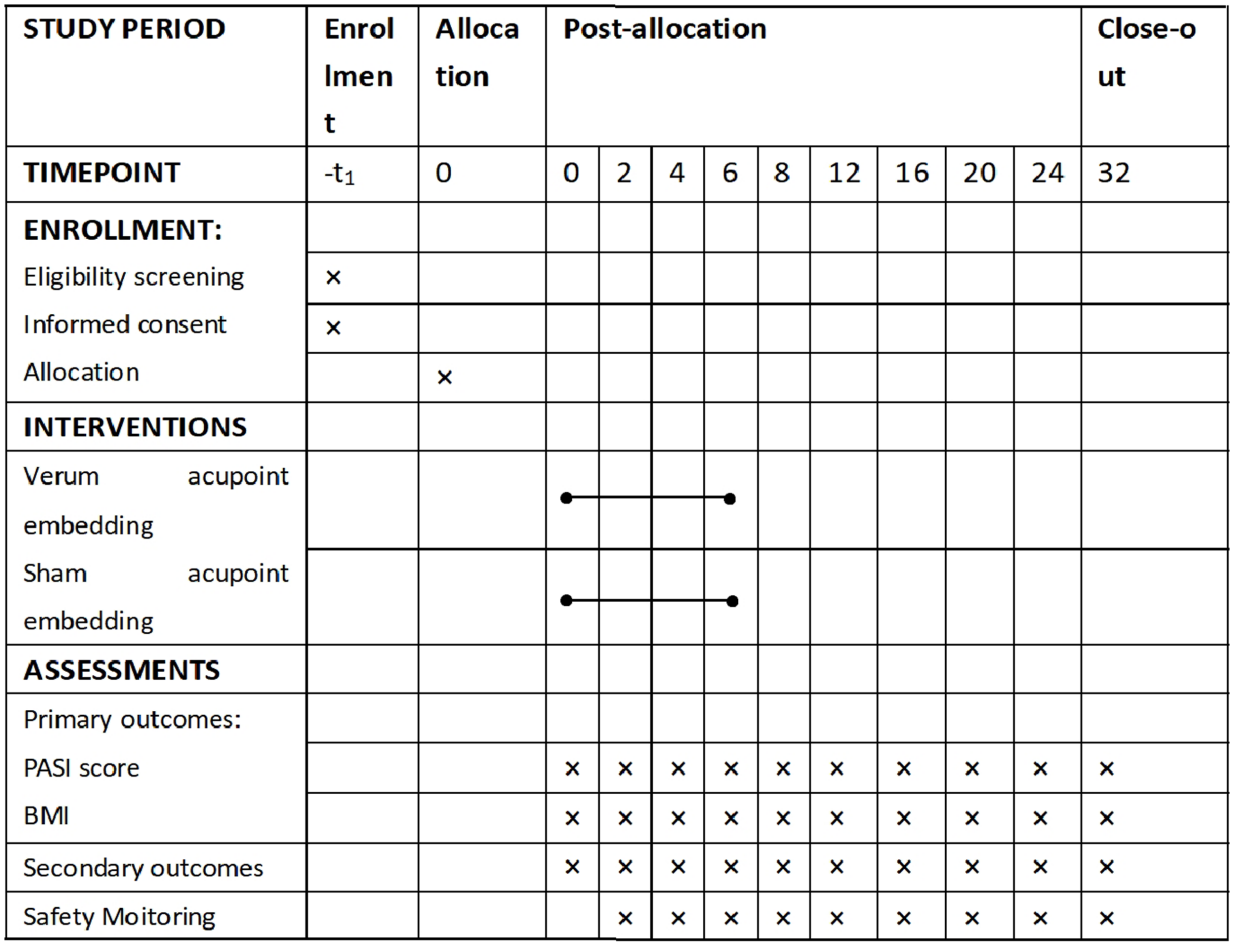
Participant timeline.

The schedule of enrollment, interventions, and assessments is summarized in Figure 5.

### 2.17 Plans for assessment and collection of outcome

To reduce costs, all research institutes will undergo unified training, including tasks such as data collection and data calculation. All experiments will be conducted by a single operator.

1 Measurement of psoriasis-related outcome indicators

PASI, BSA, PGA, DLQI, and itch VAS score are widely used outcome measures in psoriasis research (44–47). Their measurement methods are as follows:

(1) *PASI* (48): the PASI score will be assessed using the standard PASI scoring system. Two independent, uniformly trained investigators will perform the evaluations separately, and the average score will be calculated.
(2) *BSA*: BSA involvement will be evaluated by two independent, uniformly trained investigators. The palmar surface of one hand (including fingers) will be considered as 1% of the total BSA. The investigators will assess the affected area separately, and the average value will be recorded.
(3) *PGA*: the PGA will be performed by physicians, who will comprehensively evaluate the participants’ psoriatic lesions based on erythema, scaling, and infiltration (thickness). The assessment will utilize a 0–4 point scale, with lower scores indicating milder disease severity.
(4) *DLQI* (49): the DLQI will be assessed using the standard DLQI questionnaire. Study participants will complete the questionnaire under investigator guidance to evaluate the impact of psoriasis on their quality of life.
(5) *Itch VAS score (VAS)*: the VAS will be used to assess participants’ subjective itching sensation. Participants will rate their itch intensity on the VAS scale.
2 Body weight and metabolism-related indicators and assessment methods

After the expert panel discussed within our research team, the following parameters will be included in the assessment: BMI, WC, hip circumference, WHR, blood pressure, blood lipids, and blood glucose (50–53). The measurement methods are as follows:

(1) *BMI* (54): BMI will be calculated as weight (kg) divided by height squared (m^2^), following measurement of participants’ height and weight. BMI categories (kg/m^2^) are defined as: underweight (< 18.5), normal (18.5–23.9), overweight (24–27.9), and obesity (≥ 28).
(2) *WC*: WC will be measured using a non-elastic tape. Participants will stand upright with feet 25–30 cm apart, weight evenly distributed, shoulders and abdomen relaxed, and breathing normally. The measurement will be taken at the midpoint between the lower margin of the last palpable rib and the top of the iliac crest (approximately 0.5–1.0 cm above the umbilicus), with the tape horizontal and snug but not compressing the skin. A WC of ≥ 80 cm in women and ≥ 85 cm in men will indicate possible abdominal obesity, while a WC of ≥ 85 cm in women and ≥ 90 cm in men will indicate central obesity.
(3) *Hip circumference (HC)*: HC will be measured using a non-elastic tape. Participants will stand upright with feet together, arms at their sides, and breathing normally. The measurement will be taken at the point of greatest gluteal protrusion.
(4) *WHR*: WHR will be calculated as WC divided by HC. Normal values are defined as ≤ 0.85 for men and ≤ 0.80 for women.
(5) *Blood pressure*: blood pressure will be measured by trained medical personnel using a sphygmomanometer. The average reading from both arms will be recorded. Hypertension associated with obesity is defined as ≥ 140/90 mmHg. The types and doses of antihypertensive medications used by participants will be documented.
(6) *Lipid profile*: laboratory analyses, including triglycerides (TG), low-density lipoprotein cholesterol (LDL-C), high-density lipoprotein cholesterol (HDL-C), and total cholesterol (TC), will be ordered by physicians and performed by the hospital laboratory. The types and doses of lipid-lowering medications used by participants will be recorded.
(7) *Blood glucose*: laboratory analyses, including fasting blood glucose (FBG), glycated hemoglobin (HbA1c), and serum insulin, will be ordered by physicians and performed by the hospital laboratory. The types and doses of glucose-lowering medications used by participants will be recorded.
(8) *Serum uric acid*: serum uric acid levels will be measured by the hospital laboratory. The types and doses of uric acid-lowering medications used by participants will be recorded.

3 Safety indicators and assessment methods

Complete blood count, liver function, and renal function: laboratory tests including complete blood count, liver function, and renal function were ordered by physicians and performed by the hospital laboratory.

(1) *ECG*: ECG was ordered by physicians and performed by the hospital ECG department.
(2) *AEs*: AEs were reported by study participants during the intervention and follow-up periods, regardless of their relationship to the study intervention.

All data collection time points are shown in Tables 1, 2.

### 2.18 Retention strategies

In order to keep the participants involved for as long as possible, we implemented a comprehensive approach which included among others: a logistical support (transportation subsidies, flexible scheduling, and reminders via SMS/phone/WeChat), active engagement (regular health education, a dedicated app for calorie tracking, and personal progress feedback), and a system of rewards (compensation for time and travel at every visit). For those who dropped out, an early termination visit was organized to obtain primary/secondary efficacy outcomes (with consent), safety data, reasons for withdrawal, and final medication records. Data from lost to follow-up participants who had done at least two assessments were considered and counted in the intention-to-treat (ITT) analysis.

### 2.19 Outcome

#### 2.19.1 Primary efficacy endpoints

We selected the PASI and BMI as primary endpoints, as they are well-established metrics for psoriasis lesions and obesity, respectively. Through expert panel discussion, a consensus-based decision was made to define the primary outcomes as the absolute change in PASI improvement score and the absolute change BMI at Week 8. The formulas for calculation are presented below:

(1) PASI improvement rate at week 8 = (baseline PASI score - PASI score at Week 8) / baseline PASI score × 100%.
(2) Change in BMI at week 8 = baseline BMI - BMI at Week 8.

#### 2.19.2 Secondary outcomes

After discussion, the experts reached the following conclusion:

1 Disease severity (assessed at weeks 4 and 8, unless specified)

(1) Improvement rates: PASI (Week 4 only), BSA, and TCM Syndrome score.
(2) Calculated as: (baseline score - score at time point) / baseline score.

2 Metabolic and inflammatory parameters (change from baseline; Δ = baseline - week 4/8 value)

These types of parameters include: erythrocyte sedimentation rate (ΔESR), C-reactive protein (ΔCRP), body weight (ΔWeight), waist circumference (ΔWC), waist-to-hip ratio (ΔWHR), body mass index (∆BMI at Week 4 only),lipids and glucose metabolism.

3 Subgroup analyses (change from baseline in participants with abnormal values at baseline)

Additionally, a subgroup analysis was prespecified for patients with abnormal baseline metabolic parameters or metabolic syndrome. For these patients, changes from baseline in HbA1c, fasting serum insulin, uric acid, blood pressure, and lipid levels will be assessed at Weeks 4 and 8. This analysis is in line with methodologies described in previous literature. The changes will be calculated as follows:

Difference of blood pressure (Δsystolic BP) (55): In participants with baseline > 120/90 mmHg (Weeks 4, 8).
Difference of fasting plasma glucose (ΔFPG): In participants with baseline > 6.1 mmol/L (Weeks 4, 8).
Difference of glycated hemoglobin (ΔHbA1c): In participants with baseline > 6.0% (Weeks 4, 8).
Difference of uric acid (∆UA): In participants with baseline > 416 μmol/L (men) or > 358 μmol/L (women) (Weeks 4, 8).

We will collect electronic data through Excel spreadsheets and use double data entry to validate key efficacy variables in real time, with data administrators reviewing data quality reports weekly. Only attending physicians, researchers participating in the clinical study, and monitors will have access to participants’ personal records, and they will handle them confidentially. Data will be processed anonymously, omitting any personally identifiable information of participants, and personal privacy, such as facial images, will be anonymized. Participants’ personal information will be stored in the hospital’s laboratory system and medical records. Researchers should preserve all research materials, including confirmations from all research participants (which can effectively verify different records, such as original hospital records), all original informed consent forms signed by research participants, detailed records of all clinical forms, and so on. Researchers should retain clinical trial data for 5 years after the termination of the clinical research work.

### 2.20 Quality control

To ensure standardization and consistency in study implementation and data collection, all research personnel will undergo systematic and standardized training. The training curriculum will cover the following key areas: participant identification and screening procedures, standardized ACE operation protocols, methods for evaluating efficacy and safety outcomes, identification and management procedures for AEs, and standardized completion of case report forms. Furthermore, an independent quality monitoring committee consisting of three experts will be established to oversee trial conduct and data security throughout the study. This committee will be responsible for identifying and reporting any protocol deviations or adverse events in a timely manner and may recommend pausing the trial if necessary, thereby safeguarding trial quality and protecting participant rights to the greatest extent possible.

### 2.21 Statistical methods

All participants who provided written informed consent and completed baseline assessments will be included in the primary analysis, consistent with the ITT principle. Data will be analyzed according to the group to which participants will be initially randomized, regardless of treatment adherence, protocol deviations, or loss to follow-up, to minimize selection bias and reflect real-world clinical effectiveness. For missing data, a stratified strategy will be adopted: preference will be given to filling in missing values using existing data through multiple imputation or mixed-effects models, while also conducting per-protocol set analyses (including only participants with ≥ 80% compliance) as sensitivity verification; for categorical data, if the proportion of missing values is ≤ 5%, the indicator will be directly excluded, and if it is > 5%, the FIML method will be used.

All statistical analyses will be performed using a pre-specified statistical analysis plan. The primary analysis will use a linear mixed-effects model for repeated measures to analyze the changes from baseline in the continuous PASI score and BMI at week 8. As these will be pre-specified co-primary endpoints evaluating distinct therapeutic dimensions, no adjustment for multiple will be applied. Each endpoint will be tested independently at a two-sided significance level of *α* = 0.05. A similar approach will be used for continuous secondary outcomes (e.g., DLQI, WC, lipid profiles, inflammatory markers) will be presented as mean ± standard deviation for continuous variables and as frequencies (n) and percentages (%) for categorical variables. Within-group comparisons (e.g., baseline vs. Week 8) will be analyzed using paired t-tests for normally distributed data or Wilcoxon signed-rank tests for non-normally distributed data. Between-group comparisons will be performed using independent samples t-tests or Mann–Whitney U tests. Categorical outcomes will be compared using Chi-square tests (or Fisher’s exact tests). A two-sided *p*-value < 0.05 will be considered statistically significant. All analyses will follow the intention-to-treat principle. For exploratory outcomes assessed at extended time points (Weeks 12, 16, 24, and 32), the analysis will be primarily descriptive. Data are presented as mean ± standard deviation (or median with interquartile range for non-normally distributed data). No formal between-group hypothesis testing will be performed for these time points, as the data will be intended to illustrate long-term trends rather than confirm treatment efficacy. All statistical tests will be two-sided with a significance level of *α* = 0.05.

Pre-specified subgroup analyses will be performed to explore the heterogeneity of treatment effects on the primary outcomes. Interactions between treatment group and the following baseline covariates will be tested using the linear mixed model by including an interaction term: sex (male, female), age (< 45 years, ≥ 45 years), baseline BMI (< 28 kg/m^2^, ≥ 28 kg/m^2^), baseline disease duration (< 5 years, ≥ 5 years)

Analysis of AEs will be performed on the safety population, summarizing the incidence of adverse events and changes in laboratory parameters by treatment group using descriptive statistics. Formula: AEs rate (%) = (number of participants with a treatment-related adverse reaction / total number of participants receiving the treatment) × 100%. The results of subgroup analyses will be interpreted as exploratory.

No adjustment for multiple comparisons will be made for these analyses.

The analysis will handle missing data using a complete-case approach without imputation.

### 2.22 Harms

During the study, local pain, swelling, distension, and itching may occur at the acupoint embedding sites, and adverse changes such as decreased appetite, diarrhea, and constipation may also occur (56–59).

To reduce these injuries, we will strictly screen participants before enrollment. If relevant symptoms already appear, we will systematically assess their severity: (1) If there is local redness and pain, no special treatment is required; keep the area dry, and if the pain is significant, cold compresses can be applied. (2) If local subcutaneous bleeding occurs, apply a cold compress to stop the bleeding, followed by warm compresses to promote bruise absorption. (3) If sutures are expelled, disinfect with iodine solution and avoid pulling them out by yourself; if accompanied by redness and swelling, medical treatment is required. (4) If local induration occurs, it often resolves on its own after surgery; if the induration continues to enlarge, medical treatment is necessary. (5) If allergic reactions or shock should occur, stop the experiment immediately and seek medical treatment as appropriate.

### 2.23 Protocol version

The complete trial protocol (Version 1, dated August 20, 2025) is provided as a supplementary document with this submission and will be freely accessible in the published study protocol. If the protocol needs to be revised, the clinical trial registry will be updated to reflect the revision. The version control of the protocol document will be strictly maintained, and all participating sites will be required to use the most recent, approved version.

The main findings of this trial will be submitted for publication in a peer-reviewed scientific journal. Additionally, the primary results will be uploaded to a public clinical trial registry to ensure timely public access. To translate the research into practical benefit, a lay summary of the results will be prepared for trial participants and disseminated through relevant patient advocacy groups. Furthermore, the validated intervention protocol will be promoted through public health education and academic conferences.

## 3 Discussion

Psoriasis is recognized as a systemic inflammatory disorder frequently exacerbated by visceral adiposity. Adipose tissue functions as a pro-inflammatory reservoir, secreting cytokines such as TNF-*α* and IL-6 that upregulate the Th17/IL-17/IL-23 axis (60, 61). Because traditional topical therapies fail to address this systemic driver, our trial employs a “double-hit” strategy via Acupoint Catgut Embedding (ACE). This approach aims to simultaneously resolve cutaneous lesions and underlying metabolic dysfunction, effectively disrupting the metabolic-inflammatory loop.

ACE is distinguished from manual acupuncture by its capacity for sustained stimulation. While manual needling provides only transient modulation, the embedding of absorbable sutures induces prolonged mechanical and chemical stimulation for up to 2 weeks, which is thought to be essential for remodeling chronic inflammatory and metabolic pathways (62, 63). This therapeutic effect is further directed by acupoint specificity; for instance, stimulation of ST25 (Tianshu) has been associated with the regulation of lipid metabolism in clinical studies (64–67). Such sustained, multi-target modulation addresses the crosstalk between immune and metabolic systems, where ameliorating insulin resistance and systemic cytokine loads is hypothesized to facilitate the resolution of psoriatic plaques (66, 67).

Compared with biological therapies, ACE represents a pragmatic, integrative option for specific patient populations. While IL-17 and IL-23 inhibitors are highly effective for moderate-to-severe psoriasis, their application in mild cases is often limited by cost–benefit considerations and regulatory indications. Additionally, evidence regarding the impact of biologics on metabolic comorbidities remains inconsistent across clinical trials. ACE offers a cost-effective alternative with a favorable safety profile, particularly for patients with mild psoriasis who are not candidates for systemic biologics but remain impacted by the inflammatory burden of excess body weight. By targeting both adiposity and systemic inflammatory markers, ACE provides a comprehensive management strategy tailored to this metabolic-cutaneous phenotype.

Our selection criteria were carefully dictated by both methodological rigor and ethical feasibility. We specifically enrolled patients with mild psoriasis (PASI < 3 or BSA < 3) because the trial design required a baseline topical treatment of only simple emollients (urea ointment). Excluding moderate-to-severe cases was an intentional ethical decision to avoid interfering with the intensive standard-of-care therapies those patients require, which would have confounded the specific therapeutic signal of ACE. Furthermore, the BMI inclusion threshold was set at ≥ 24 kg/m^2^ to ensure recruitment feasibility; a more restrictive cutoff (e.g., ≥ 30 kg/m^2^) would have unnecessarily limited the participant pool and underpowered the study without changing the underlying biological premise of metabolic-driven inflammation.

The methodological strengths of this trial are characterized by its multicenter framework and the implementation of specific protocols designed to mitigate performance bias—a persistent challenge in acupuncture-related blinding. By utilizing pre-loaded delivery devices and third-party randomization, we prioritized internal validity to ensure the reliability of the therapeutic signal. Beyond its rigorous design, this study addresses a significant gap in existing literature by incorporating an extended 32-week follow-up phase. This longitudinal perspective is essential for evaluating whether metabolic adjustments are sustained and how they influence long-term disease recurrence patterns. Furthermore, the integration of high-resolution molecular analyses—including single-cell sequencing and proteomics—allows for a granular investigation into the systemic crosstalk between metabolic health and cutaneous immune homeostasis. By moving beyond clinical observation, these exploratory findings may elucidate the biological pathways through which ACE modulates the psoriasis-obesity phenotype, offering a mechanistic foundation for integrative therapeutic strategies.

## 4 Limitation

Although the PASI-based sample size calculation is more directly aligned with the primary endpoint of psoriasis, the absence of robust sham-controlled RCT data means that estimates derived solely from PASI assumptions, rather than BMI-based parameters, may still carry inherent uncertainty. Despite the use of a rigorous blinding design (such as sham embedding needles and independently prepared tool kits by a third party), potential bias from the operating physicians may still influence the results. In addition, although the sham embedding group simulated the needle insertion and pushing actions, there is an inherent difference from real embedding in terms of the physical stimulation of “thread implantation,” which may affect the equivalence of the placebo effect. We acknowledge the potential confounding effects of concomitant medications. Although we required patients to maintain stable regimens for underlying comorbidities throughout the trial, the complex interplay of medications commonly used in this population may still influence weight and inflammatory outcomes. As an exploratory trial, this study did not implement a drug washout period or perform subgroup analyses based on specific medication classes. Future studies should incorporate longer baseline observation periods or include medication usage as covariates to better delineate the treatment effect of acupoint catgut embedding. In addition, the primary endpoint of this study was set at week 8, with extended follow-up for secondary endpoints up to week 32. However, the sustainability of long-term efficacy after short-term intervention still requires further research, especially considering the chronic nature of psoriasis and the risk of weight rebound.

## Glossary

MetS: Metabolic syndrome
ACE: Acupoint catgut embedding
RCTs: Randomized controlled trials
DLQI: Dermatology Life Quality Index
PASI: Psoriasis Areaand Severity Index
BSA: Body Surface Area
VAS: Verbal Analogue Scale
WC: Waist circumference
BMI: Body Mass Index
WHR: Waist-to-hip ratio
PGA: Physician Global Assessment
ESR`: Erythrocyte sedimentation rate
CRP: C-reactive protein
HC: Hip circumference
LDL-C: Low-density lipoprotein cholesterol
BP: Blood pressure
TG: Triglycerides
HDL-C: High-density lipoprotein cholesterol
FBG: Fasting blood glucose
HbA1c: Glycated hemoglobin
ECG: Electrocardiogram
AEs: Adverse events
